# Decrease of voriconazole trough levels during therapy with enteral nutrition: a case report

**DOI:** 10.1186/s40780-021-00237-4

**Published:** 2022-02-03

**Authors:** Hiromi Kaneko, Shingo Yamazaki, Masashi Uchida, Takaaki Suzuki, Kentaro Murakami, Hisahiro Matsubara, Katsuhiko Kamei, Itsuko Ishii

**Affiliations:** 1 Division of Pharmacy, University Hospital, Chiba University Hospital, 1-8-1 Inohana, Chuo-ku, Chiba City, Chiba, 260-8677 Japan; 2 Department of Frontier Surgery, Graduate School of Medicine, Chiba University, 1-8-1 Inohana, Chuo-ku, Chiba City, Chiba, 260-8670 Japan; 3 Medical Mycology Research Center, Chiba University, 1-8-1, Inohana, Chuo-ku, Chiba City, Chiba, 260-8673 Japan

**Keywords:** Voriconazole, Enteral nutrition, Jejunostomy tube feeding, Therapeutic drug monitoring, Drug interaction

## Abstract

**Background:**

Voriconazole (VRCZ) is the first-line therapy for chronic pulmonary aspergillosis and is available in both intravenous and oral formulations. The bioavailability of the oral form is estimated to be over 90% in healthy volunteers. Some drugs are reported to interact with enteral nutrition (EN), but there are few reports about the trough levels of VRCZ during EN therapy. Here, we describe changes in the VRCZ trough levels in a patient receiving continuous EN therapy.

**Case presentation:**

The patient was a 58-year-old man with esophageal cancer and a history of partial pulmonary resection due to aspergilloma. He was taking oral VRCZ tablets and his VRCZ trough level was about 2 μg/mL before esophageal cancer surgery. Following esophagectomy, VRCZ was restarted on postoperative day 16. Crushed VRCZ tablets were administered via a jejunostomy tube because of swallowing difficulty. He was also receiving EN, which was interrupted only during the administration of VRCZ. When we checked his VRCZ level 5 days after restarting VRCZ, the trough level was 0.80 μg/mL. After increasing the VRCZ dose, reducing EN, and changing the administration route from jejunostomy tube to oral, his trough level increased to 1.87 μg/mL.

**Conclusions:**

A decrease in the VRCZ trough level was observed when VRCZ was administered via a jejunostomy tube while the patient was receiving continuous EN. Careful monitoring of VRCZ levels is needed in such cases.

**Supplementary Information:**

The online version contains supplementary material available at 10.1186/s40780-021-00237-4.

## Introduction

Voriconazole (VRCZ), an antifungal triazole, is the first-line therapy for chronic pulmonary aspergillosis [1]. It is available in both intravenous and oral formulations, and oral therapy is a useful option for outpatients.

VRCZ is metabolized mainly by cytochrome P450 (CYP) 2C19 and to a lesser extent by CYP3A4 and CYP2C9 [2]. Poor metabolizers achieve 4-fold higher VRCZ levels than extensive metabolizers [3]. VRCZ trough levels below 1.0 μg/mL are associated with lack of response to therapy [4]. On the other hand, high trough levels are associated with serious toxicity such as neurotoxicity and hepatotoxicity [5]. Hamada et al. reported that an appropriate trough level for increasing clinical success while minimizing toxicity is 1.0–4.0 μg/mL [6], and therefore therapeutic drug monitoring is needed.

The absorption of VRCZ tablets is a first-order process, and the bioavailability exceeds 90% according to data from healthy volunteers [7]. VRCZ bioavailability is reduced by approximately 20% when administered with high-fat meals [8]. Although administration between meals is indicated in the package insert [3], it is difficult to administer VRCZ between meals in patients who are receiving continuous enteral nutrition (EN). Early EN reduces the incidence of life-threatening surgical complications [9], and an EN strategy is obviously important for those who cannot take food orally. However, some drug interactions with continuous EN have been reported [10].

Williams reported that continuous EN via a naso-jejunal catheter for 24 h caused a decrease in the trough level of orally administered VRCZ from therapeutic levels to undetectable levels [11]. Although VRCZ levels when administered via a tube have been reported [12, 13], the backgrounds of the patients differed and detailed information on EN was not described.

Here, we describe the temporal changes in VRCZ trough levels in a patient with esophageal cancer before and after starting continuous EN therapy over the entire clinical course.

## Case

The patient was a 58-year-old Japanese man with stage III (T3N3M0) esophageal cancer who was scheduled for neoadjuvant chemotherapy and surgery. He had a history of partial pneumonectomy for chronic pulmonary aspergillosis and required antifungal agent therapy to avoid relapse of the pulmonary aspergillosis.

**Fig. 1:**
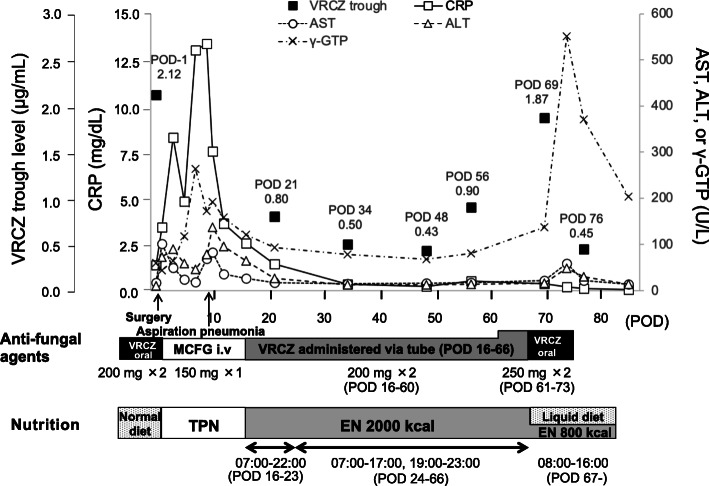
Perioperative data relating to anti-fungal treatment and nutritional strategy for this patient. VRCZ trough levels, C-reactive protein and change in liver function values in the perioperative period are shown in relation to the timing of anti-fungal agent regimen and nutritional strategy. VRCZ, voriconazole; MCFG, micafungin; POD, postoperative day; i.v., intravenous injection; TPN, total parenteral nutrition; EN, enteral nutrition; CRP, C-reactive protein; AST, aspartate transferase; ALT, alanine aminotransferase; γ-GTP, γ-glutamyl transpeptidase

VRCZ tablets were initiated at a dose of 200 mg (3.8 mg/kg) twice daily at 06:00 and 18:00. At days 27 and 41 after starting VRCZ, trough levels were 1.93 μg/mL and 2.46 μg/mL, respectively. The only other concomitant drug in the preoperative period was magnesium oxide (Table 1). The patient received five courses of chemotherapy before surgery. During chemotherapy, the VRCZ tablets were changed to micafungin (MCFG) injections because he could not tolerate oral drugs due to nausea. After the end of chemotherapy, the VRCZ tablets were re-introduced. Sixteen days later, on the day before surgery, the VRCZ trough level was 2.12 μg/mL and the C-reactive protein (CRP) value was 1.39 mg/dL (Fig. 1).

**Table 1:** List of drugs administered concomitant with VRCZ oral tablets in this case

Drug administered	Dose	Frequency
Preoperative period		
Magnesium oxide	500 mg	TID
Postoperative period		
Lansoprazole	15 mg	SID
Sodium chloride	2.5 g	TID
Mosapride citrate hydrate	5 mg	TID
Rikkunshito extract	2.5 g	TID
Bromhexine hydrochloride	4 mg	TID
Acotiamide hydrochloride hydrate	100 mg	TID

*TID* three times a day, *SID* once a day

The patient underwent right thoracic esophagectomy and jejunostomy. There was an increase in CRP to 13.38 mg/dL on postoperative day (POD) 9, due to aspiration pneumonia, which decreased over time. The CRP value was 2.57 mg/dL on POD 16. EN with Hine E-Gel® was started the same day. The administration method was 15 h continuous during the daytime, from 07:00 to 22:00. Hine E-Gel® contains low-molecular peptides and amino acids and the proportion of fat is 20.3% (Table 2). From the day of the operation to POD 15, oral VRCZ administration was changed to MCFG injections. On POD 16, VRCZ was restarted via a jejunostomy tube. VRCZ tablets were crushed and suspended in water. The VRCZ dosing regimen was the same as before the esophagectomy: 200 mg twice daily at 06:00 and 18:00. On PODs 16–23, at 18:00, the administration of EN was stopped just before the administration of VRCZ. The line was flushed with water, VRCZ was administered immediately, and the line was again flushed with water. EN was restarted soon after. All concomitant drugs in the postoperative period are listed in Table 1. On POD 21, the VRCZ trough level was 0.80 μg/mL and the CRP value was 1.44 mg/dL (Fig. 1). There were no episodes of severe diarrhea, which could have caused malabsorption and changes in body weight. The jejunostomy tube was inserted 25 cm from ligament of Treitz and a check of the positioning revealed no apparent problems.

**Table 2:** Nutritional content of Hine E-Gel® started for enteral nutrition in this case

Composition	Content (/100 mL)	Energy to total calories (%)
Calorie	80 kcal	
Protein	3.2 g	16.4
Fat	1.8 g	20.3
Carbohydrate	12.3 g	63.2
Dietary fiber	1.1 g	
Sodium	133 mg	

We considered the possibility of an interaction between VRCZ and EN and temporarily stopped EN 1 h before and 1 h after VRCZ administration from POD 24. On PODs 24–66, EN was clamped at 17:00, 1 h before VRCZ administration at 18:00. After flushing the line with water, VRCZ was administered immediately, and the line was again flushed with water. EN was started at 19:00, 1 h after the administration of VRCZ. However, the trough level was 0.50 μg/mL on POD 34 (Fig. 1). When examined again on POD 48 and POD 56, the levels were still under the therapeutic range at 0.43 μg/mL and 0.90 μg/mL, respectively (Fig. 1). We confirmed that his adherence was good and there were no episodes of tube obstruction. The patient’s general status was relatively good, and the CRP value was 0.21 mg/dL on POD 48. On POD 56, CRP had increased to 0.51 mg/dL, which might be explained by an aspiration episode. However, this was not too severe and the patient soon recovered. The duration of interruption to EN could not be extended further because faster administration caused diarrhea.

On POD 61, VRCZ was increased to 250 mg (4.9 mg/kg) twice daily, administered at 06:00 and 18:00. On POD 67, the administration route was switched from jejunostomy tube to oral after stable swallowing function was confirmed. At the same time, the patient resumed oral feeding and EN was reduced from 15 h continuous infusion to 8 h continuous infusion, from 08:00 to 16:00. This allowed a 2-h interval between EN and VRCZ administration. On POD 69, 2 days after EN was reduced, the trough level was 1.87 μg/mL (Fig. 1). The CRP value on POD 69 was 0.37 mg/dL. At the same time, an increase in the γ-glutamyl transpeptidase (γ-GTP) level was observed. On POD 73, the γ-GTP increase reached grade 3 according to the Common Terminology Criteria for Adverse Events version 5.0 [14] and VRCZ administration was discontinued. The VRCZ trough level 3 days after stopping VRCZ was 0.45 μg/mL. The γ-GTP level decreased at the same time. We decided against VRCZ rechallenge and were continuing to monitor the patient for signs of a recurrence of fungal infection.

VRCZ levels were measured by high-performance liquid chromatography with minor modifications of the method of Pennick et al. [15] All blood samples for the measurement of VRCZ trough levels were collected before 06:00. Plasma VRCZ was extracted using a solid extraction column (ISOLUTE® SLE+, Biotage, Japan). The analytical column was a CAPCELL PAK C18 MG (5 mm, 250 × 4.6 mm, Osaka Soda, Japan), and the ultraviolet wavelength for VRCZ was 257 nm. The minimum limit of quantitation was 0.25 μg/mL.

## Discussion and conclusion

After esophagectomy, we observed that the VRCZ trough level was decreased to below the therapeutic level on POD 21, 5 days after VRCZ was restarted. Among the concomitant drugs administered in the postoperative period (Table 1), lansoprazole is metabolized by the same enzymes as VRCZ. Fang reported that VRCZ levels increased when combined with lansoprazole [16]. However, there are no reports on the concomitant drugs used in our case that could affect VRCZ metabolism and cause a decrease in serum levels.

From POD 16, the patient received continuous EN therapy. Table 3 summarizes the previously reported effects of EN therapy on the levels of VRCZ administered orally or via a tube. Williams reported that oral VRCZ trough levels decreased to undetectable levels in a patient receiving continuous EN therapy with Isosource® HN, an EN formula composed of 29% fat [11]. There are two similarities between our case and Williams’ case: the EN used contained a relatively high proportion of fat (20.3% in our case) and it was administered continuously. On the other hand, crushed VRCZ tablets were administered via a jejunostomy tube in our case, whereas VRCZ suspension was orally administered in Williams’ case. Two other studies on VRCZ levels after administration via a tube have reported that the levels were in the therapeutic range [12, 13]. However, no detailed information was given on EN status, such as composition, calories, and duration. Taken together, we consider that tube administration itself is a useful route and that continuous EN with a relatively high fat content seems to have played an important role in the decrease in VRCZ trough levels in our case.

**Table 3:** Summary of previously reported effects of enteral nutrition on the levels of VRCZ administered orally or via a tube

	Williams^**11**^	Martinez et al^**12**^	Mohammedi et al^**13**^
**Study type**	Case report	Case report	Prospective, observational, single-center study
**VRCZ dose**	11.6 mg/kg/day	8 mg/kg/day	6.2 ± 1.1 mg/kg/day
**VRCZ formulation, Administration route**	Suspension, Per oral	Crushed VRCZ tablets, Jejunostomy tube	Crushed VRCZ tablets, Nasogastric tube
**Nutritional route**	Nasojejunal tube	NR	Nasogastric tube
**Type of EN (composition)**	ISOSOURCE® HN (29% caloric content of fat)	NR	NR
**EN method**	Continuous	NR	Interrupted only for the duration of VRCZ administration
**Patients, Age**	1 post-transplantion, age 13 years	1 with esophageal cancer, age 66 years	6 with hematological malignancies (4 acute leukemia, 2 myeloma), 1 with solid tumor, and 1 with invasive pulmonary aspergillosis, age 63 ± 12 years
**Patients, Race**	Hispanic	NR	NR
**The purpose of VRCZ therapy**	Prophylactic therapy	*Candida grabrata* infection	4 patients, voriconazole therapy was given for a microbiologically documented fungal infection. In total, five fungal pathogens were isolated (4 *Aspergillus fumigatus* and 1 *Candida kefyr*).
**Serum VRCZ levels (days after starting tube administration)**	1.07 mg/dL decreased to undetectable (On day8, 15)It was not mentioned that whether these were trough levels or not.	Ctrough: 1.7, 1.75, 1.4 mg/L Cpeak: 2.5, 2.55, 2.6 mg/L (On days 2, 8, 28)	Ctrough: 4.6 ± 2.8 mg/L Cpeak: 6.4 ± 4.3 mg/L(On days 3–44, mean 16 days)
**Assay method**	NR	Solid-phase extraction followed by reversed liquid phase chromatography with UV detection.	Solid-phase extraction followed by reversed liquid phase chromatography with UV detection

NR, data not reported

On days 24–66, we tried stopping the EN between 17:00 and 19:00 for VRCZ administration at 18:00, but its trough levels remained below the therapeutic range. The time to peak concentration of VRCZ varies by 1.7–3.0 h in patients at high risk of developing fungal infections [17]. Hence, a 1-h interval might not have been sufficient for our patient to avoid an interaction with EN.

On PODs 16–60, the VRCZ trough levels varied from 0.43 μg/mL to 0.90 μg/mL. There was no change in the concomitant drugs administered or in liver function. Although inflammation, reflected by CRP levels, has been reported to be associated with VRCZ trough levels [18], there was no apparent increase in CRP during this period. Therefore, we consider the changes in VRCZ trough levels on PODs 21, 34, 48, and 56 might have been caused by intra-individual variability.

The VRCZ trough level on POD 69 increased from 0.90 μg/mL to 1.87 μg/mL. On POD 61, the VRCZ dose was increased from 200 mg (3.9 mg/kg) to 250 mg (4.9 mg/kg). On POD 69, there was no increase in CRP level and no other significant episodes that would cause a decrease in VRCZ clearance. We simulated the VRCZ levels using VFEND® TDM tool version 1.2 (Pfizer, Japan). When the observed trough levels in our case were used, the predicted trough level at steady state after increasing the dose from 200 mg to 250 mg was approximately 1.0 μg/mL (Additional file 1). The actual VRCZ trough level was 1.87 μg/mL and higher than that predicted. Therefore, this increase in the VRCZ trough level on POD 69 was more likely caused by a recovery of VRCZ absorption rather than increased dose.

On POD 67, the route of VRCZ administration was changed from tube to oral. Given that the jejunostomy tube was 25 cm from the ligament of Treitz, we consider that there was enough length for the absorption of drugs. In an in vitro study, it has been reported that the recovery rate after infusion with VRCZ suspension through an enteral feeding tube is 99.3 ± 10.3% after the tube is flushed twice with 20 mL of distilled water [19]. This indicates that the change in the route of administration of VRCZ had little effect on the increased VRCZ level on POD 69. On POD 67, EN was also reduced from 2000 kcal to 800 kcal and a 2-h interval was instituted between VRCZ and EN administration. We believe that this reduction in EN caused recovery of VRCZ absorption and the increase in VRCZ trough level.

The duration of VRCZ trough levels below 1.0 μg/mL on PODs 21–56 corresponded to when the patient received continuous EN therapy with Hine E-Gel®. Therapeutic drug monitoring is therefore essential to maintain the appropriate VRCZ trough levels. In the case of severe infection, it might be advisable to switch from oral VRCZ tablets to injections in patients receiving continuous EN therapy.

In conclusion, even though the oral formulation of VRCZ has high bioavailability, VRCZ levels should be monitored carefully when the patient’s EN status changes.

## Supplementary Information

**Additional file 1.** Bayesian-predicted concentration curve of VRCZ. The mean values of the default population pharmacokinetic parameters for VRCZ in VFEND® TDM tool version 1.2 (Pfizer, Japan) were as follows; Ka, 0.654 /h; Km, 3.850 mg/L; Distribution Volume of central compartment, 97.9 L; Vmax, 29.631 mg/h; F, 1.000; lag-time, 0.190 h. The individual VRCZ level of days after dose escalation was predicted by the Bayesian method. The observed VRCZ trough levels during enteral nutrition were used for the analysis. The VRCZ dose was increased from 200 mg every 12 h to 250 mg every 12 h on POD 61, and the predicted VRCZ trough level 9 days after the dose increased (POD 69) was 1.00 μg/mL.

## Data Availability

Not applicable.

## Abbreviations

VRCZ: Voriconazole
EN: Enteral nutrition
CYP: Cytochrome P450
MCFG: Micafungin
POD: Postoperative day
CRP: C-reactive protein
γ-GTP: γ-glutamyl transpeptidase
i.v.: Intravenous injection
TPN: Total parenteral nutrition
AST: Aspartate transferase
ALT: Alanine aminotransferase
