# Supplementary material for: Decrease of voriconazole trough levels during therapy with enteral nutrition: a case report
Source: J Pharm Health Care Sci. 2022 Feb 3;8:6. doi: 10.1186/s40780-021-00237-4 (PMC8812174; doi:10.1186/s40780-021-00237-4)
Supplement: Supplementary file 1 — Additional file 1. Bayesian-predicted concentration curve of VRCZ. The mean values of the default population pharmacokinetic parameters for VRCZ in VFEND® TDM tool version 1.2 (Pfizer, Japan) were as follows; Ka, 0.654 /h; Km, 3.850 mg/L; Distribution Volume of central compartment, 97.9 L; Vmax, 29.631 mg/h; F, 1.000; lag-time, 0.190 h. The individual VRCZ level of days after dose escalation was predicted by the Bayesian method. The observed VRCZ trough levels during enteral nutrition were used for the analysis. The VRCZ dose was increased from 200 mg every 12 h to 250 mg every 12 h on POD 61, and the predicted VRCZ trough level 9 days after the dose increased (POD 69) was 1.00 μg/mL. [file 40780_2021_237_MOESM1_ESM.pdf]

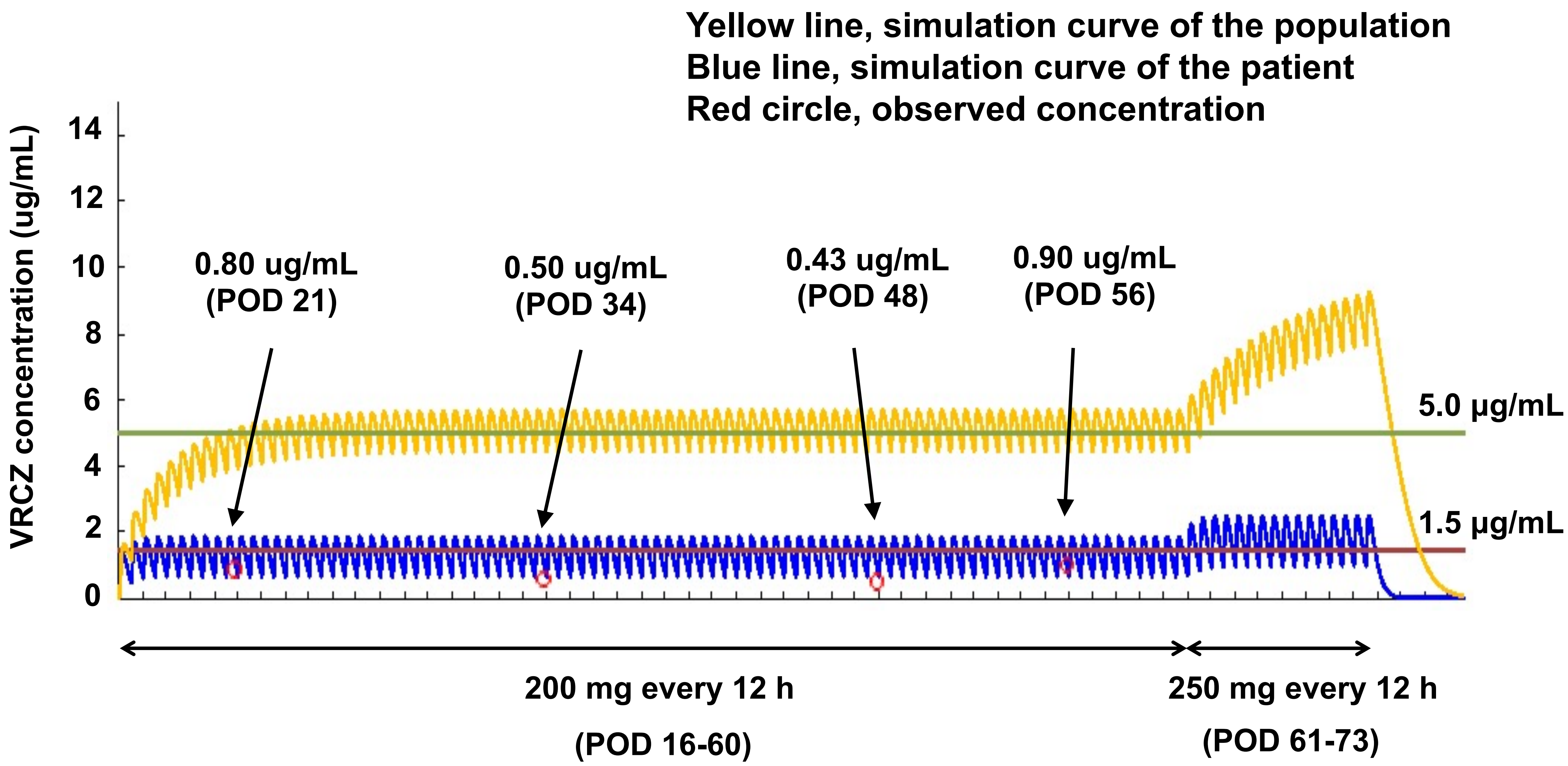
Yellow line, simulation curve of the population
Blue line, simulation curve of the patient
Red circle, observed concentration
VRCZ concentration (ug/mL)
14
12
10
8
6
4
2
0
0.80 ug/mL
(POD 21)
0.50 ug/mL
(POD 34)
0.43 ug/mL
(POD 48)
0.90 ug/mL
(POD 56)
5.0 μg/mL
1.5 μg/mL
200 mg every 12 h
(POD 16-60)
250 mg every 12 h
(POD 61-73)
